# Portable electroanalytical nucleic acid amplification tests using printed circuit boards and open-source electronics†

**DOI:** 10.1039/d2an00923d

**Published:** 2022-08-15

**Authors:** Anna Toldrà, Alar Ainla, Shirin Khaliliazar, Roman Landin, Georgios Chondrogiannis, Martin Hanze, Pedro Réu, Mahiar M. Hamedi

**Affiliations:** School of Engineering Sciences in Chemistry, Biotechnology, and Health, KTH Royal Institute of Technology Stockholm 10044 Sweden mahiar@kth.se; International Iberian Nanotechnology Laboratory 4715-330 Braga Portugal

## Abstract

The realization of electrochemical nucleic acid amplification tests (NAATs) at the point of care (POC) is highly desirable, but it remains a challenge given their high cost and lack of true portability/miniaturization. Here we show that mass-produced, industrial standardized, printed circuit boards (PCBs) can be repurposed to act as near-zero cost electrodes for self-assembled monolayer-based DNA biosensing, and further integration with a custom-designed and low-cost portable potentiostat. To show the analytical capability of this system, we developed a NAAT using isothermal recombinase polymerase amplification, bypassing the need of thermal cyclers, followed by an electrochemical readout relying on a sandwich hybridization assay. We used our sensor and device for analytical detection of the toxic microalgae *Ostreopsis cf. ovata* as a proof of concept. This work shows the potential of PCBs and open-source electronics to be used as powerful POC DNA biosensors at a low-cost.

## Abstract 2

We have repurposed industrial, standard printed circuit boards (PCBs) for DNA sensing and integrated them with an open-source portable potentiostat. This work paves the way towards low cost, miniaturized electronic systems for on-site testing.

## Graphical abstract

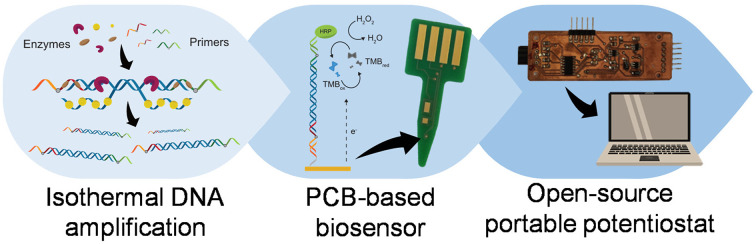

## Introduction

Nucleic acid amplification tests (NAATs) are very sensitive and specific molecular techniques with broad application in disease diagnosis, health surveillance, food safety, and environmental monitoring.^1,2^ Their implementation, however, currently requires trained personnel and expensive instrumentation only available in modern centralized facilities, resulting in a time lag between sample procurement and analysis,^3^ and high cost. Centralized PCR tests have certainly played a crucial role in global public health during the ongoing SARS-CoV-2 pandemic.^4^ The utilization of the PCR tests during this pandemic has also made it clearer that “one size does not fit all” with regards to public health testing, and that point-of-care (POC) tests with much higher dissemination can provide valuable information and significantly contribute to public health.^5^

The realization of a low-cost and automated POC NAAT is however challenging, as it requires many steps including: sample preparation with sample collection and DNA extraction,^6,7^ DNA amplification,^8,9^ and DNA detection.^10,11^ Each of these steps including their subparts must be simplified and minimized for integration into POC devices. Portable, over-the-counter NAATs available in the market for home use are still not widely utilized. Additionally, the majority of NAATs commonly use PCR for amplification followed by a fluorescent or colorimetric readout.

To eliminate the need for thermocycling and facilitate its integration into portable devices, several isothermal techniques have been developed.^8,12^ Among these, recombinase polymerase amplification (RPA) may be the most promising as it is fast (20–40 min), operates at low temperatures of 37 to 42 °C and uses a simple experimental design.^13^ RPA has been coupled with different detection methods including colorimetric,^14–17^ fluorescent,^18–20^ and electrochemical readouts.^21–24^

Electrochemical DNA biosensors appear well suited for POC testing since they facilitate high level miniaturization, compatibility with microfluidic systems, and integration into portable devices, thus precluding the utilization of optical parts such as those found in qPCR thermocycles or in gel-doc instruments.^2,25^ In addition, they combine the specificity of the DNA hybridisation event with a highly sensitive, rapid, quantitative measurement.^25,26^ Given these outstanding advantages, an increasing number of electrochemical DNA biosensors have been developed to date.^27–32^ However, two reasons may still be hampering their dissemination: (i) the use of expensive/non-standardized electrodes, and (ii) the lack of real portability/miniaturization. Regarding the latter, the readout of electrochemical signals generally depends on expensive benchtop instruments.^21–24^ Nevertheless, the rapid development of electronics and software has enabled new potentiostats. Apart from the expensive commercially-available portable potentiostats (*e.g.* Sensit Smart), we^33^ and others^34–37^ have developed inexpensive, open-source and portable potentiostats that can carry out common electrochemical techniques at the point of need.

Current electrode fabrication involves complex and non-standardized processes and/or the use of expensive substrates. Electrodes for DNA sensing are commonly made of gold because it allows the formation of well-defined self-assembled monolayers (SAM) using thiol-modified DNA probes.^38^ Printed circuit boards (PCBs), originally designed for mounting electronic components, have features which render them appealing to be repurposed for micro-total-analysis systems, resulting in “lab-on-PCBs”.^39,40^ Although the lab-on-PCB approach was first suggested in the late 1990s,^41,42^ it has recently re-emerged^39,40,43^ because two reasons: (i) digital electronics and communications are ubiquitous today, and (ii) chips and standard PCBs have become very cheap. These factors enable both the rapid, low-cost prototyping, as well as scalable industrial mass manufacturing of electronics. Additionally, the substrates, mostly polyimide or glass fiber composites, are inexpensive and inert to liquids, allowing microfluidics to be mounted on-top of PCBs. The printing resolution can be as low as 100 μm and many layers can be stacked to form complex 3D electrodes. Furthermore, PCBs offer the possibility to be easily integrated into sensors, actuators, and electronics. The interest of using PCBs as electrodes for DNA sensing is growing,^44–48^ but no device has been yet applied to the detection of isothermally amplified DNA.

Here we demonstrate the capacity of custom-designed, mass-produced PCBs to act as disposable electrodes for DNA sensing, and its subsequent integration with a custom-designed, portable, open-source potentiostat designed for DNA detection. As a proof-of-principle, we identified DNA of the toxic microalgae *Ostreopsis cf. ovata* after RPA amplification using tailed primers and a sandwich hybridization assay (SHA) using a capture probe immobilized on the PCB electrode for electrochemical detection. This work shows the potential of PCBs and standard open-source electronics to be used as powerful portable platforms for on-site NAAT sensing at a low-cost.

## Experimental

### Materials

Pierce™ TMB enzymatic substrate kit for horseradish peroxidase (HRP) detection (containing a solution of 0.4 g L^−1^ 3,3′,5,5′-tetramethylbenzidine (TMB) and a solution of 0.02% H_2_O_2_) were bought from Thermo Fisher Scientific (Sweden). 24 K pure eco gold was acquired from Gold Plating Services (USA). TwistAmp Basic RPA kit was purchased from TwistDX Limited (UK) and QIAquick PCR Purification Kit from QIAGEN (Germany). Custom DNA oligonucleotides (Table S1†) were synthetized by Biomers (Germany). All other reagents were purchased from Sigma Aldrich (Sweden).

### PCB design and fabrication

**Fig. 1:**
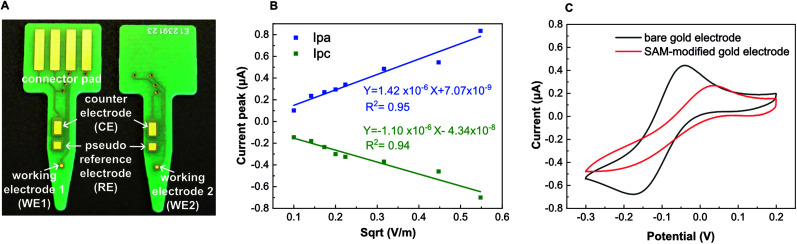
Design and electrochemical characterization of the PCBs. (A) Each PCB has 4 gold electrodes. (B) Randles–Sevcik plots of bare gold electrodes and SAM-modified gold electrodes after conducting CVs in 5 mM ferricyanide at different scan rates (10 mV s^−1^–300 mV s^−1^) (*n* = 3). (C) Representative CV in 5 mM ferricyanide (scan rate of 30 mV s^−1^) after gold electroplating: bare gold electrode and SAM-modified gold electrode.

The PCBs were designed using the Eagle 9.6.2 (Auto-desk Inc., CA, USA) software. We designed 4 gold electrodes: one counter (2.6 mm^2^ on each side, 5.2 mm^2^ in total), one reference (1.44 mm^2^ on each side, 2.8 mm^2^ in total) and two working (0.25 mm^2^ each) electrodes. The 3-electrode system is symmetrical on both sides of the PCB (Fig. 1A). PCBs were manufactured by Eurocircuits N.V. (Belgium) following a standard PClass6 process for two-layer PCB with Che Ni/Au surface finish. They have a thickness of 1.55 mm and are composed of FR4 glass fiber epoxy composite in the middle part, both sides covered with an array of copper (35–50 μm), nickel (3–6 μm) and gold (65–100 nm) layers. Except for the electrodes and connector, the device is covered with epoxy-based solder mask. PCB electrodes have contact pads fitted for standard card edge connectors (166087-4, TE Connectivity, Switzerland) to allow for electrical connection.

### Gold electroplating

The PCBs were cleaned by a 15 min immersion in a solution containing Milli-Q, 0.5 M potassium hydroxide and 30% H_2_O_2_ (5 : 1 : 1) followed by 5 min sonication in a sequence of acetone, ethanol and Milli-Q, and finally air-dried. An alkaline non-cyanide 24K pure eco gold solution was used for gold electroplating. A 2-electrode configuration was used: stainless steel as anode (3 cm^2^) while all 4 electrodes of the PCBs as cathode (16.5 mm^2^). The PCB was soaked in 30 mL of gold-plating solution kept at 50 °C under mild agitation. The plating was carried out by applying a 3.2 mA cm^−2^ cathodic current density, which corresponds to a current of 272 μA (cathodic surface area was equal to 16.5 mm^2^) for 11 min. After electroplating, PCBs were rinsed with Milli-Q for 1 min and air-dried. Electroplating was performed with a Cromocol potentiostat (Bio-Logic Scientific instrument, Scandinavia).

### Characterization and functionalization of the electrode surface

Gold electroplated electrodes were cleaned as previously described (Gold electroplating section). Cyclic voltammograms (CVs) were performed in the 3-electrode set up in 5 mM ferricyanide (K_3_[(FeCN)_6_]) solution by cycling the potential between −0.3 and +0.2 V at different scan rates (10 mV s^−1^–300 mV s^−1^).

Cleaned PCBs were functionalized with a thiolated DNA capture probe. Each PCB was immersed in 70 μL (volume to cover only the two working electrodes) of 500 nM capture probe in PBS to allow the formation of the SAM. After incubation at 4 °C for at least 16 h, PCBs were rinsed with PBS containing 5% Tween 20 (PBS-Tween), then with Milli-Q, and finally air-dried. Blocking of active sites was performed by immersion in 70 μL of 100 μM 6-mercapto-1-hexanol (MCH) in Milli-Q for 30 min at room temperature under shaking. After each step, the PCBs were rinsed again and air-dried. CVs were performed between −0.3 and +0.2 V at different scan rates (10 mV s^−1^–300 mV s^−1^) in 5 mM ferricyanide solution. CVs were performed using a Cromocol potentiostat (Bio-Logic Scientific instrument, Scandinavia).

### Potentiostat design and fabrication

A detailed description and design files of the potentiostat can be found in ESI (Fig. S1–S7 and Tables S1–S5†). The potentiostat has the following four features: (i) 3-electrode configuration; (ii) available potential range −2.9 to +0.4 V (resolution: 0.85 mV, non-linearities: 1.6 mV); (iii) single current range from −20 to +10 μA (sensitivity: 2.9 nA, noise: 6.9 nA, non-linearities <100 nA at <0.3% of FS); and (iv) support for common electrochemical techniques: chronoamperometry (CA), cyclic voltammetry (CV) and square-wave voltammetry (SWV).^33^

Circuit diagram and main components of the potentiostat are depicted in Fig. S1, S2 and S3.† The electronics are based on microcontroller ATMega168 (Microchip Technologies), which communicated with computer over serial bus (UART), interfaced through USB-to-serial converter (FT232). Microcontroller has integrated analog-to-digital converter (ADC) for signal sampling and two external 12-bit digital-to-analog converters (DACs) to dictate the electrode potential. DACs are controlled over I2C serial bus from the microcontroller. The potentiostat is based on quadruple operational amplifier (OA) LM2902DRG4 (Texas Instruments). The microcontroller was programmed in C language using Arduino IDE. Data is acquired over serial (COM) port in Windows computer, and the data acquisitions and user interface software were written using Microsoft Visual Studio, .NET framework and programming language C Sharp. The potentiostat has a size of 2.3 × 7 cm and it includes an on/off switch (Fig. S5†) that enables to precisely record current output after 1 s.

### Nucleic acid amplification test (NAAT)

The NAAT involves a first step of RPA in solution and a second step of a SHA on the SAM-modified PCB electrode with electrochemical detection. The primers and probes used in this work were previously reported^24^ and are listed in Table S1.† Primers were specific for *O*. cf. *ovata* and were modified with tails. Probes for the SHA were complementary to these tails: a thiolated capture probe and an HRP-labelled reporter probe.

RPA reactions were performed at 37 °C for 30 min in a low-cost and portable thermal block following previously optimized parameters.^24^ Briefly, each RPA reaction (50 μL) contained: 22.95 μL of nuclease-free water, 14.75 μL of rehydration buffer, ½ lyophilized enzyme pellet, 2.4 μL of 10 μM of tailed primer, 2.5 μL of 480 mM magnesium acetate and 5 μL of DNA, which corresponded to a positive sample (1 pM of target synthetic DNA) or a blank (nuclease-free water). The efficiency of RPA reactions was confirmed through 3% agarose gel electrophoresis. RPA products were stored at −20 °C until use.

SHA was performed on SAM-functionalized PCBs (Characterization and functionalization of the electrode surface section) in two steps: (i) incubation in a 70 μL solution containing 40 μL of RPA product and 30 μL of PBS, and (ii) incubation in 70 μL of 10 nM HRP-labelled reporter probe in 2% w/v skimmed milk in PBS. Both steps were carried out at room temperature for 30 min with shaking and, after each step, PCBs were first rinsed with PBS-Tween, then with Milli-Q and finally air-dried.

In the following step, PCBs were incubated with 200 μL (volume to cover the 3-electrode set up) of TMB/H_2_O_2_ enzymatic substrate and allowed to react for 3 min. Finally, CA measurements of the enzymatically generated TMB in its oxidized form was carried out by applying a reducing potential of −0.2 V for 1 s and then reading the current output. CVs in TMB/H_2_O_2_ showed that a potential lower than −0.1 V induced the complete reduction of TMB (Fig. S8†). A working potential of −0.2 V was chosen for CA measurements as we obtained the best discrimination between blank and positive samples.

CA measurements were performed using: (i) an Autolab PGSTAT204N with MUX 16 module (Metrohm Autolab, Sweden) with the accompanying NOVA 1.11 software package, and (ii) the homemade open-source portable potentiostat. For each PCB, both working electrodes (WEs) were used: first the WE1 and, after 30 s, the WE2. Results are presented as mean ± standard deviation (SD) for five independent samples (*n* = 5). Statistical analysis between two groups was conducted using unpaired Student's *t*-test through SigmaStat Software, considering a *p*-value ≤0.05.

## Results and discussion

Our rationale for the design paradigm is to utilize and repurpose the industrial electronic manufacturing standard of PCBs for the realization of near-zero cost sensors as disposable parts, in conjunction with design and fabrication of very low-cost portable multiuse detectors (potentiostats). The potentiostat's cost at scale (1000 units) would be 8.48 EUR/unit (Table S2†). The total cost of the test including PCB (0.14 EUR for 10 000 units), PCB modification (0.072 EUR) and assay reagents (1.89 EUR) would be 2.1 EUR/test. It is worth noting that these prices are expected to be even lower in the future as electronics and, most importantly, bioassay reagents become cheaper.

We designed the PCB to function as an electrochemical sensor with 4 electrodes: two small working electrodes, a counter electrode and a quasi-reference electrode (Fig. 1A). We designed small working electrodes to reduce cost, to facilitate miniaturization and because these have been reported to perform better than macro-electrodes.^49^ We manufactured the PCB platforms following a standard industrial protocol having a metal trilayer consisting of copper (35 μm), nickel (3–6 μm) and emulsion gold (65–100 nm). The metallic layers were subsequently covered with industrial standard epoxy-based solder mask, which here acted as a perfect liquid barrier. We left the parts of the electrode surface open to allow liquid contact. The gold layer of standard commercial PCBs is around 65–100 nm, which was not thick enough to produce reproducible electrochemical measurements. Although a thicker gold layer can also be included during the industrial manufacturing of PCBs,^48^ it increases its price drastically and it is difficult to order at a small scale. In this study, we therefore, cleaned their surface and subsequently modified them by conducting an in-house gold electroplating (Experimental section) to achieve a gold layer with a thickness of roughly 2 μm according to the manufacture specifications.^44,46^ However, these previous studies did not show detection of isothermally amplified DNA on PCB, which we are the first to report here. After cleaning the gold-electroplated surface, we performed CVs in 5 mM ferricyanide solution at different scan rates. The linear and symmetrical Randles–Sevcik plots (Fig. 1B) obtained between the peak current and the square root of the scan rate demonstrated a reversible (fast) electron transfer only controlled by diffusion, with an effective surface area of 0.04 mm^2^ for the gold working electrode. To evaluate the capacity of PCBs to support SAMs using thiolated DNA capture probes, we conducted CVs in the presence of 5 mM ferricyanide solution, as it is a well-stablished methodology to evaluate SAM functionalization.^50–52^ CVs recorded in the presence of ferricyanide demonstrated a successful immobilization of the capture probes as seen by the change in the electron transfer between bare and SAM-modified gold working electrodes (Fig. 1C). A diagram of the surface chemistry is presented in Fig. S9.†

**Fig. 2:**
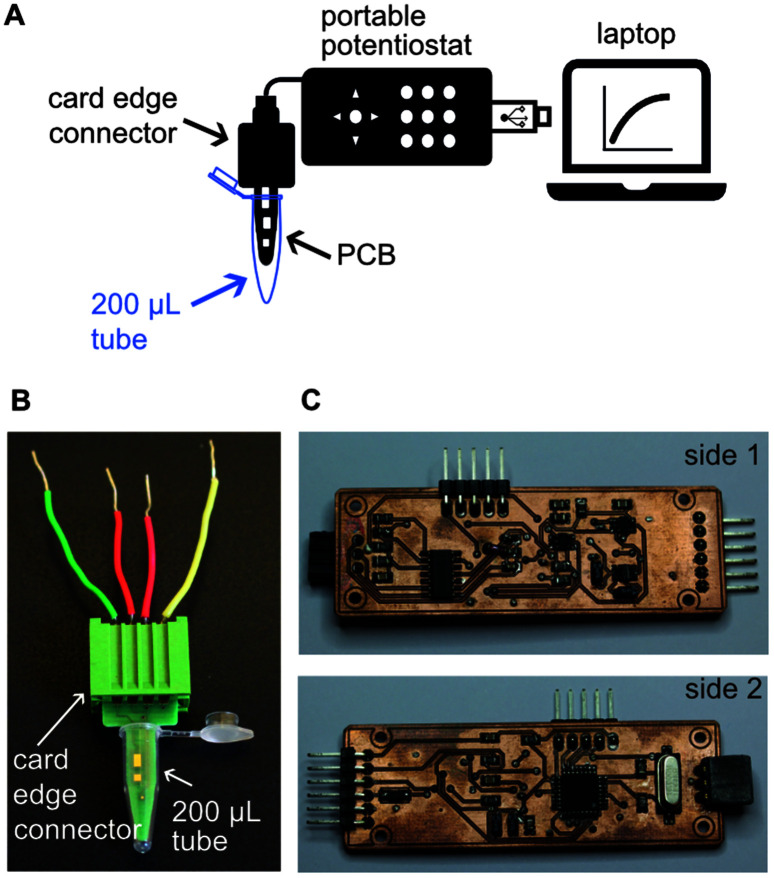
Schematics and pictures of the electroanalytical device. (A) Schematics of the potentiostat showing connection with the computer and the PCB for sample analysis. (B) Picture of the PCB with a card edge connector, fitting inside a 200 μL tube. The size of a PCB is 1.2 × 3.1 cm. (C) Picture of the main components of the potentiostat. The size of the potentiostat is 2.3 × 7 cm.

Schematics and pictures of the PCB showing the connection with the potentiostat and the computer for sample analysis are depicted in Fig. 2A. The PCB design had one end fitting into a 200 μL PCR tube for liquid handling while conducting the assay (Fig. 2B). The other end of the PCB sensors fitted a card edge connector to enable connection with our portable open-source potentiostat. Our in-house designed open-source portable potentiostat (Fig. 2C) has following measurement characteristics: (i) potential range −2.9 to 0.47 V with 0.85 mV resolution, and (ii) current range: −22 to 12 μA with 2.8 nA resolution, 6.9 nA noise and accuracy 0.3% of full-scale (detailed description of the potentiostat can be found in ESI†). Furthermore, the potentiostat is plugged into a computer's USB port and has a power consumption of 50 mW, making it extremely suitable for implementation in low-resource settings. Future devices can easily integrate batteries and digital communication in the device to make it free-standing and controllable through smart phones without any significant increase in cost or complexity, and we have indeed already shown this capability.^33^

**Fig. 3:**
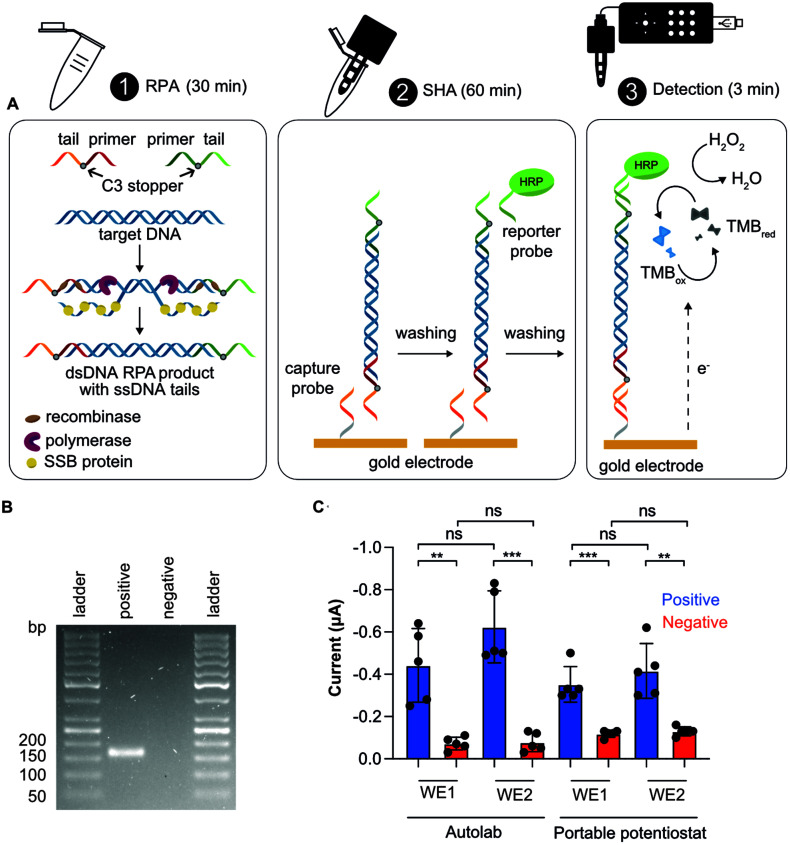
Schematics and results of the NAAT. (A) Schematics of the RPA followed by a SHA with electrochemical detection: (1) RPA is performed in solution. The RPA product is a dsDNA sequence with specific ssDNA tails at each end (due to the use of tailed primers); (2) the RPA amplicon hybridizes with the capture probe. After a washing step, the HRP-labelled reporter probe hybridizes with the RPA product; and (3) once TMB/H_2_O_2_ enzymatic substrate is added, reduction of TMB is measured by chronoamperometry. (B) Representative gel electrophoresis confirming the successful RPA amplification of the tailed target (148 bp dsDNA + 35 bp ssDNA). (C) Chronoamperometric results using Autolab and the portable potentiostat for positive (1 pM target synthetic DNA) and blank (nuclease-free water) samples for gold working electrode 1 (WE1) and 2 (WE2). Chronoamperometry aggregated data showing a statistically significant between groups (*n* = 5) at *p*-value ≤0.05 (unpaired student's *t* test).

We coupled our NAAT on PCBs with our open-source portable potentiostat to perform the measurements. This NAAT involves a first step of RPA in solution followed by a SHA on the PCB substrate with electrochemical readout, as illustrated in Fig. 3A. We used isothermal RPA because it operates at low and constant temperature and is there-fore easy to integrate into POC devices. We performed RPA reaction in solution for positive (1 pM target synthetic DNA) and blank (nuclease-free water) samples, and exploited tailed-modified primers for the RPA reaction. These consisted of a single-stranded DNA (ssDNA) sequence (“tail”) added to the primer, where the primer and tail are separated by a 3-C alkyl chain preventing elongation of the tail during amplification resulting in dsDNA products with ssDNA tails at each end. Gel electrophoresis results confirmed a successful RPA amplification of the tailed target (148 bp dsDNA + 35 bp ssDNA) (Fig. 3B).

We utilized the SHA for DNA detection because of its simplicity, as RPA products can be directly detected using probes complementary to the tails without the need for post-amplification steps such as ssDNA generation,^53–55^ and because of its specificity, as it involves two hybridization steps. This assay includes three steps: (i) the working electrode is functionalized with the capture probe, blocked with MCH and incubated with the RPA product, which hybridizes with the capture probe; (ii) the HRP-labelled reporter probe is added to hybridize with the complementary tail; and (iii) the TMB/H_2_O_2_ enzymatic substrate is added for chronoamperometry.

We conducted CA measurements for NAATs including positive and blank samples, using both an Autolab and our portable potentiostat. The results presented in Fig. 3C show significant discrimination between positive and blank samples both using the Autolab and the portable potentiostat, thus demonstrating the utility of the PCB electrode combined with our potentiostat for DNA sensing. Finally, we measured signals from both working electrodes (WE1 and WE2) on the PCB sensor: first the WE1 and, after 30 s, the WE2. Although current means were higher for WE2 than WE1 (there was a time-lag of 30 s), they were not significantly different (Fig. 3C), showing the potential of our system to do multiplex analysis. Repeatability (intra-day precision) and reproducibility (inter-day precision) were appropriate, with relative standard deviation (RSD) values of 12.4 and 16.5%, respectively, for positive samples and WE1. Given the low cost of the PCB electrodes (*i.e.* 0.14 EUR for 10 000 units), we present them as a disposable platform. Nevertheless, we successfully showed that PCBs can be re-used at least here times by performing a cleaning step after detection (Fig. S10†), also showing no degradation due to edge effects.^48^

The target concentration that we tested (1 pM) corresponds to 1500 microalgal cells/reaction, provided that one cell of *O*. cf. *ovata* has 2137 ribosomal DNA copies per cell.^56^ This is therefore a relevant concentration for application purposes, since it allows quantifications below the current alarm thresholds proposed for *Ostreopsis* cells (10 000–30 000 cells per L seawater).^57^ Our previous electrochemical biosensors have shown excellent LODs and specificity using both synthetic^58^ and genomic DNA targets,^24^ besides being successfully applied to environmental samples.^24^ Moreover, the biosensors did not require any purification after the amplification step,^24,55^ as also demonstrated for other electrochemical biosensors based on RPA and a SHA.^59^ The device presented here has the potential to reach the same assay analytical characteristics and, more importantly, move towards miniaturized and portable diagnostic devices at low cost.

## Conclusions

Here we show that industrial standard PCBs can be re-purposed to act both as near-zero cost electrodes for SAM-based DNA biosensing, as well as for the fabrication of a low-cost potentiostat to enable electrochemical readout from these PCB electrodes. To show the analytical capability of this system, we developed a NAAT using isothermal RPA amplification, bypassing the need of thermal cyclers, followed by an electrochemical readout relying on a sandwich hybridization assay. The operational time of the PCB-based DNA sensor, without sample pre-treatment step, was 93 min and required three steps in total: (i) RPA (30 min); (ii) sandwich hybridization assay (60 min); and (iii) electrochemical detection (3 min).

To the best of our knowledge, this is the first report that combines isothermal DNA amplification, PCB technology and an inexpensive and portable potentiostat. PCBs facilitate mass production of electrodes at a low-cost, and the use of open-source electronic devices systems allows for iterative improvement by crowdsourcing, towards integrated, and miniaturized systems with low power consumption. Future work will include molecular identification of other relevant DNA biomarkers, as well as subsequent assay characterization and validation. Ongoing research should focus on three specific areas: (i) shortening of the incubation times of the sandwich hybridization assay as well as performing stability studies of the SAMs on PCBs to achieve ready-to-use platforms;^59^ (ii) integration of RPA on chip by using heating,^60,61^ by performing a solid-phase amplification,^62^ and by using microfluidics on PCBs^60^ to achieve a true lab-on-a-chip device; and (iii) inclusion of a sample preparation step on chip by exploiting magnetic beads-powered cell capture^63^ and enzymatic cell lysis and DNA extraction steps using paper.^7^ Our demonstration of SAM-based PCB sensors will likely be applicable to many other SAM-based sensors.^23,30,53,64^ Our prototype clearly demonstrates substantial superiority in terms of cost and portability over existing alternatives. We think that this open-source NAAT prototype fabricated with mass-produced PCBs and ubiquitous electronics will have an impact on addressing current commercial bottlenecks in POC diagnostic tests.

## Author contributions

AT: Conceptualization, investigation, methodology, formal analysis, writing – original draft; AA: conceptualization, software, writing – review & editing; SK: investigation; RL: software; GC: investigation, MH: investigation; PR: conceptualization, writing – review & editing; MMH: conceptualization, writing – review & editing, funding acquisition. The manuscript was written through contributions of all authors. All authors have given approval to the final version of the manuscript.

## Conflicts of interest

There are no conflicts to declare.

## Supplementary Material

AN-147-D2AN00923D-s001
