# Supplementary material for: Portable electroanalytical nucleic acid amplification tests using printed circuit boards and open-source electronics
Source: Analyst. 2022 Aug 15;147(19):4249–56. doi: 10.1039/d2an00923d (PMC9511072; doi:10.1039/d2an00923d)
Supplement: AN-147-D2AN00923D-s001 [file AN-147-D2AN00923D-s001.pdf]

Electronic Supplementary Material (ESI) for Analyst.
This journal is © The Royal Society of Chemistry 2022

Supporting Information

Portable electroanalytical nucleic acid amplification tests using printed circuit boards and open-source electronics

Anna Toldrà[a], Alar Ainla[b], Shirin Khaliliazar[a], Roman Landin[a], Georgios Chondrogiannis[a], Martin Hanze[a], Pedro Réu[a*], Mahiar M. Hamedi[a*]

[a] School of Engineering Sciences in Chemistry, Biotechnology, and Health, KTH Royal Institute of Technology, Stockholm 10044, Sweden

[b] International Iberian Nanotechnology Laboratory, 4715-330 Braga, Portugal

*Corresponding authors: Mahiar M. Hamedi (mahiar@kth.se); Pedro Réu (pedrorc@kth.se)

1. Table of Content

1. Table S1 3

2. Design of the open-source portable potentiostat 4

2.1. Acronymes 4

2.2. Hardware design 4

2.3. Firmware design 9

2.4. Computer software 11

2.5. Characterization of electrical performance 12

2.5.1. Potential control 12

2.5.2. Current measurement 13

2.6. Additional resources 15

2.7. References 15

3. Figure S8 16

4. Figure S9 17

5. Figure S10 18

1. Table S1

Table S1. List of oligonucleotide sequences and their respective modifications (underlined).

Name	Sequence (5'-3')
O. cf. *ovata* FwP with tail	gtt ttc cca gtc acg ac-C3-aca atg ctc atg cca atg atg ctt gg
Ostreopsis spp. RvP with tail	tgt aaa acg acg gcc agt-C3-gca wtt ggc tgc act ctt cat aty gt
Thiolated capture probe	gtc gtg act ggg aaa act ttt ttt ttt ttt tt-C3-SH
HRP-labelled reporter probe	HRP-act ggc cgt cgt ttt aca
O. cf. *ovata* target	aca atg ctc atg cca atg atg ctt ggt ggc atg cac ctt gtt agt tgt agc atg aca gct tga tac tta tct aaa cgc ttt cat caa ctg tct tct gac agc aat gaa tgc atc aat tca aaa caa tat gaa gag tgc agc caa atg c

2. Design of the open-source portable potentiostat

2.1. Acronyms

List of abbreviations used in this section:

- ADC – Analog-to-Digital Converter
- CA - Chronoamperometry
- CV – Cyclic Voltammetry
- DAC – Digital-to-Analog Converter
- EUR – Euro currency
- IC – Integrated Circuit
- MCU – Microcontroller Unit
- N/A – Not Applicable
- PCB – Printed Circuit Board
- SWV – Square Wave Voltammetry
- UART – Universal Asynchronous Receiver-Transmitter
- USB – Universal Serial Bus

2.2. Hardware Design

This three-electrode (working, counter, reference) potentiostat was designed to be compact, low-cost and easy to replicate and modify. We designed circuitry and PCB layout using free-version of electronics design software Eagle 9.6.2 (Autodesk Inc.). The two-layer PCB boards were fabricated using in-house engraving tool. However PCB designs can be easily submitted to any commercial PCB fabrication service (e.g. EuroCircuits, PCBWay and others.). We choose all components and made design such, that it would be readily replicated in Do-It-Yourself (DIY) manner without need for specialized or expensive equipment. All components can be ordered from common suppliers such as Mouser Inc. PCB assembly requires soldering iron with fine tip, basic soldering supplies, such as solder and flux and tweezers to handle small surface mounted (SMD) components.

Figure S1 shows the main components of the potentiostat. **Figure S2** shows the circuit diagram of the potentiostat. **Figure S3** shows the PCB layout and gives description of connectors to electrodes and USB-to-Serial interface, which was integrated as an independent module, to minimize components to be soldered. **Figure S4** shows a picture of the portable potentiostat. **Table S2** lists all components with their cost and part numbers. Total cost of components to

make the potentiostat was 8.48 EUR, which does not include the PCB board, since it was manufactured in-house.

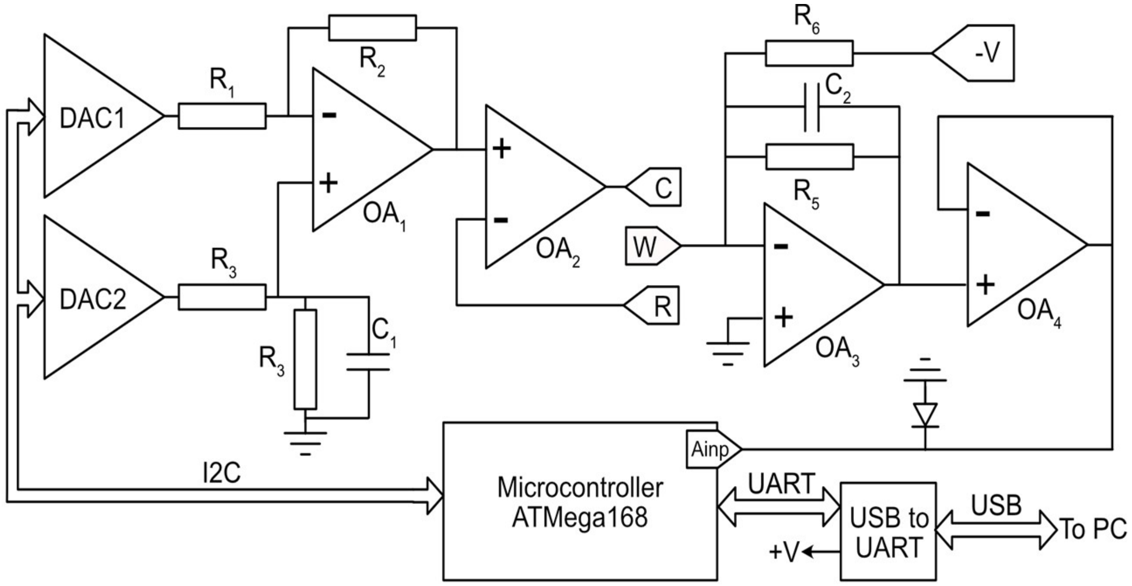

Figure S1. Picture of the main components of the potentiostat.

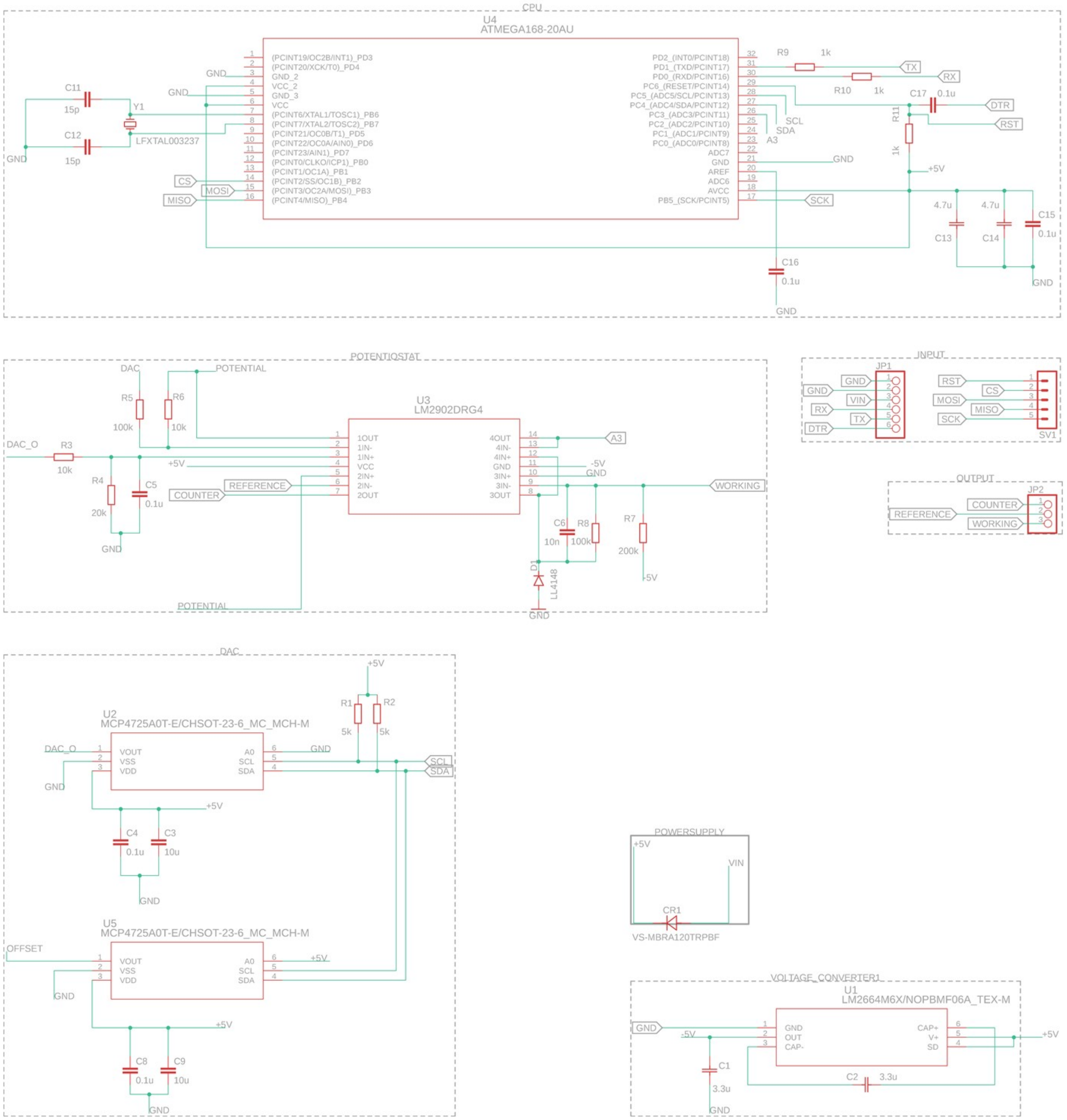

Figure S2. Circuit diagram of the potentiostat. Table S3 lists the details of components. Main components of the potentiostat are: i) microcontroller (MCU) (U4), ii) digital-to-analog converters (DACs) (U2, U5) to generate potential waveforms and iii) Quad (4x) operational amplifier (U3) for both controlling the electrode potential through feedback from the reference electrode and for trans-impedance amplifier to convert the working electrode current into voltage signal, which would be then digitalized by ADC inside of the MCU. For MCU we chose ATMEGA168 due to low-cost and compatibility with Arduino programming (Arduino Nano).

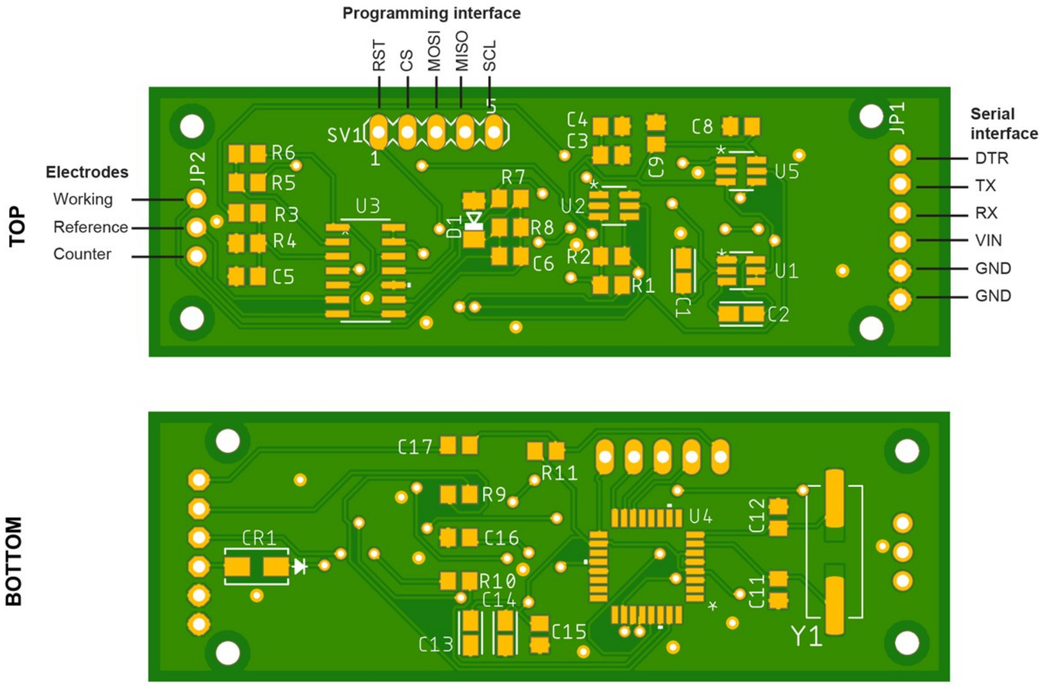

Figure S3. Layout of two-layer PCB board designed in Eagle.

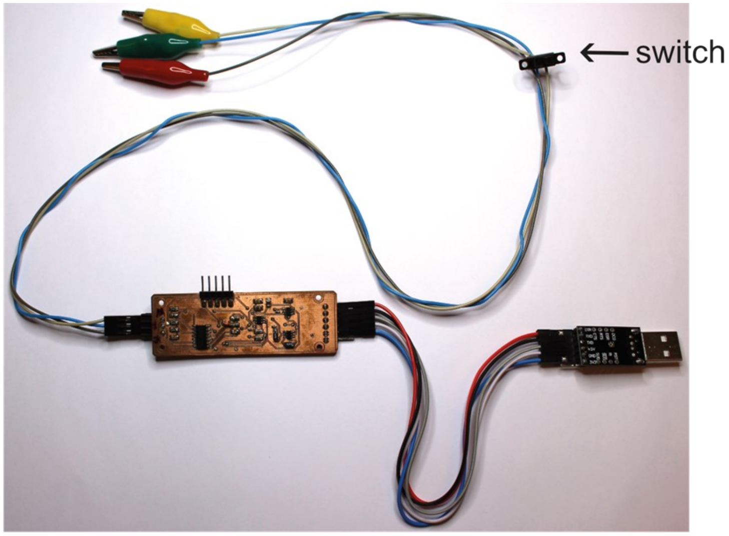

Figure S4. Picture of the portable potentiostat.

Table S2. List of components used for the construction of portable potentiostat. All part numbers and unit costs are according to Mouser catalog, except when noted otherwise. Costs are in EUR listed for manufacturing single unit (1) and larger volume of 1000 units (1k) (as of September, 2021). Total value of the bill of material for single potentiostat was 16.27EUR (excluding the PCB board, which could be ordered for 45.80EUR). Total unit cost in case of 1000 devices would be 8.48EUR including components, PCB and assembly.

Part	Value	Package	Part number	Unit cost (1)	Unit cost (1k)	Description
U1	LM2664M6/NOPB	SOT-23-6	926-LM2664M6/NOPB	0.78	0.355	Switching voltage regulator Vout=(-1)*Vin to generate -5V voltage
U2, U5	MCP4725A0T	SOT-23A	579-MCP4725A0TECH	0.93	0.775	DAC (12bit, I2C)
U3	LM2902DRG4	SOIC-14	595-LM2902DRG4	0.33	0.115	Operational amplifiers (x4)
U4	ATMEGA168-20AU	TQFP-32	556-ATMEGA168-20AU	2.71	2.58	Microcontroller
Y1	LFXTAL003237Reel	HC-49/S	449-LFXTAL003237REEL	0.43	0.213	16MHz crystal oscillator
D1	LL4148	SOD-80	512-LL4148	0.10	0.023	Diode. ADC input protection against negative potentials
CR1	SS12-E3/5AT	DO-214AC	625-SS12-E3/5AT	0.36	0.125	Diode. Protection against reversing power supply
JP1	6x1 2.54mm	Through	649-1012937990603BLF	0.14	N/A*a	Pin header. Serial communication
SV1	5x1 2.54mm	Through	649-1012937990501BLF	0.15	N/A	Pin header. AVR ICSP connector
JP2	3x1 2.54mm	Through	649-68016-403HLF	0.14	0.059	Pin header. Electrodes
		Board	895-LC234X	6.57	N/A*a	USB-to-UART bridge (See footnote *a)
R1	5k	0805	71-CRCW0805J-5K	0.13	0.019	I2C bus pull-up
R2	5k	0805	71-CRCW0805J-5K	0.13	0.019	I2C bus pull-up
R3	10k	0805	71-CRCW080510K0FKEAC	0.09	0.01	Potential offset
R4	20k	0805	652-CR0805FX-2002ELF	0.09	0.004	Potential offset
R5	100k	0805	652-CR0805FX-1003ELF	0.09	0.01	Potential slope
R6	10k	0805	71-CRCW080510K0FKEAC	0.09	0.012	Potential slope
R7	200k	0805	71-CRCW0805200KFKEAC	0.09	0.004	Potentiostat offset
R8	100k	0805	652-CR0805FX-1003ELF	0.09	0.006	Potentiostat gain
R9	1k	0805	603-RC0805FR-071KL	0.09	0.006	Microcontroller serial input
R10	1k	0805	603-RC0805FR-071KL	0.09	0.006	Microcontroller serial input
R11	1k	0805	603-RC0805FR-071KL	0.09	0.006	Microcontroller reset pull-up
C1,C2	3.3u	0805	187-CL21A335KPFNNNE	0.21	0.046	Voltage regulator capacitors
C3,C9	10u	0805	187-CL21A106KOQNNNG	0.09	0.018	Capacitors for power stabilization
C4,C5,C8, C15,C16,C17	100n	0805	80-C0805C104M3R	0.09	0.013	Capacitors for power stabilization
C6	10n	0805	710-885382207006	0.09	0.056	Defines the potentiostat bandwidth
C11,C12	15p	0805	710-885012007052	0.09	0.03	Crystal oscillator capacitors
C13,C14	4.7u	0805	187-CL21A475KOFNNNG	0.11	0.02	Microprocessor power stabilization
	FT230XQ-R	QFN-16	895-FT230XQ-R	N/A*a	1.53	USB-to-UART chip (See footnote *b)
	UP2-AH-1-TH	Through hole	490-UP2-AH-1-TH	N/A*a	0.258	USB connector (type 2.0 male) (See footnote *b)
				45.8*b	0.16*c	PCB (70.47x23.8mm), 2 layer,
				N/A	1.05*c	Assembly service

*a - For single unit USB-to-USART module was used for simplicity of assembly. For large series the module functionality could be directly integrated on board using interfacing IC (FT230) and USB connector, which would reduce overall size and cost.

*b - According to Eurocircuits.com

*c - According to PCBway.com

2.3. Firmware

Firmware was written using Arduino development environment (IDE). The firmware followed similar structure as used by us in an earlier publication about universal wireless electrochemical detector (UWED) [1], differently from UWED, which communicated with iPhone over Bluetooth, this potentiostat here was designed for computer connection via USB. USB communication was based on serial protocol, where we used USB-to-Serial interface to connect with USART port of the MCU. All commands and replies were based on ASCII text format making it easy to debug using serial console. Firmware implement simple functions to set electrode potential, perform accurately timed potential sweeps (required for CV and SWV methods) and measure working electrode current. After each measurement firmware returns results to computer in the text format. All numerical parameters used for communication with potentiostat are in digital units (digits of DAC and ADC) and require calculation on computer side to convert them from and to physical units (e.g. mV and nA) using calibration constants established for the potentiostat.

Serial communication with the potentiostat uses speed 57600 baud with no flow-control. Potentiostat operates by waiting commands from computer in format “**X(***number***)**”, where X is the command code (a capital letter in the range A-J) and number is integer parameter. After executing the command potentiostat return corresponding lower-case character and “:DONE”, e.g. command “A(2000)” would set the DAC value to 2000 (in DAC control word), after which potentiostat returns “a:done”. Some commands such as “I” (to measure electrode current) return additional numerical value after “:DONE”. When potential sweep is performed, such as CV or SWV potentiostat returns in defined time steps measured values. This communication uses following format “>*N,T,V,I*”, where N,T,V,I are integer numbers. Sequence starts with “>” symbol and values are separated by comas. N is the index of measurement in the scan, T is the timestamp of the measurement in microseconds from the beginning of the scan, V is the DAC value set as electrode potential and I is the ADC value corresponding to the electrode current.

The following **Table S3** describes all commands available for the potentiostat communication.

Table S3. Commands for the potentiostat. Char denotes the command code character.

Char	Parameters range	Function	Descriptions
A	0-4095.<br>Default: 2048	Set electrode potential	This sets the value for DAC U2, which controls the electrode potential
B	0-4095<br>Default: 4095	Set offset potential	This sets the value for DAC U5, which control potential offset. In our current design we did not alter this potential and it was kept constant throughout all measurements and tests
C	Minimum: 2500<br>Default: 10000	Set the step of the timer for sweeps in μs.	This controls the timing when potential is changed and current is measured. It is defining the frequency for SWV. SWV frequency is 1/(2*value)
D	-32768-32767<br>Default: 1	Set potential step (increment) for odd steps in the sweep	How much the electrode potential is changed in odd steps. This value is 0 for CA, it is equal with the next parameter for CV, when both steps are equal, and with opposite signs for SWV, which is composed of square wave and stair. See [1]!
E	-32768-32767<br>Default: 1	Set potential step (increment) for even steps in the sweep	Similar to previous command, but for even steps in the sweep
F	1-10000.<br>Default: 1	Set number of steps in the sweep	This is total number of steps, which will be performed in the sweep, starting from initial potential set with "A" and changing in steps defined by "D" and "E" in intervals defined by "C"
G	N/A	Start running the sweep	Start the potential sweep
H	N/A	Halt the sweep	Halt the sweep if desired
I	N/A	Perform individual current measurement	Performs the current measurement and returns the value. This is used for current monitoring in state while sweep is not performed
J	Default: 16	Set number of ADC conversions summed to form the current measurement signal	Set number of conversions to integrate. Larger the number lower is the noise, however larger number of measurements limit the measurement frequency. Current measurement calibration values need to be adjusted, when this parameter is changed.

2.4. Computer software

The computer software was developed in Visual C# language in the development environment Microsoft Visual Studio 2017 using .NET framework. Software and source code is included in the SI of this article, as well in the GitHub repository [2]. This software combines simple graphical user interface and data acquisition. Interface allows to set linear calibration parameters for the potentiostat, one pair of parameters describing the potential control (mV/dig) and another the current measurement (μA/dig) side of the system. User can choose connection mode, it can be automatic, when potentiostat is the only serial port device connected or can be chosen manually in case multiple serial port device are connected. Once device is connected user can set potential and see the measured current value. There is possibility to perform three types of measurements, chronoamperometry (CA), cyclic voltammetry (CV) and square wave voltammetry (SWV), with sets of parameters defined for each of these methods. Some methods have input text box followed by the text field. In this case the text field shows the actual applied parameter, which is closest possible to the desired parameter. This is due to the digitalization, which do not allow all parameters to be chosen exactly, but only in certain steps. Eventually user can save the data in text files, which can be easily imported to another software for further analysis. **Figure S5** shows the main view of the Potentiostat Control Application.

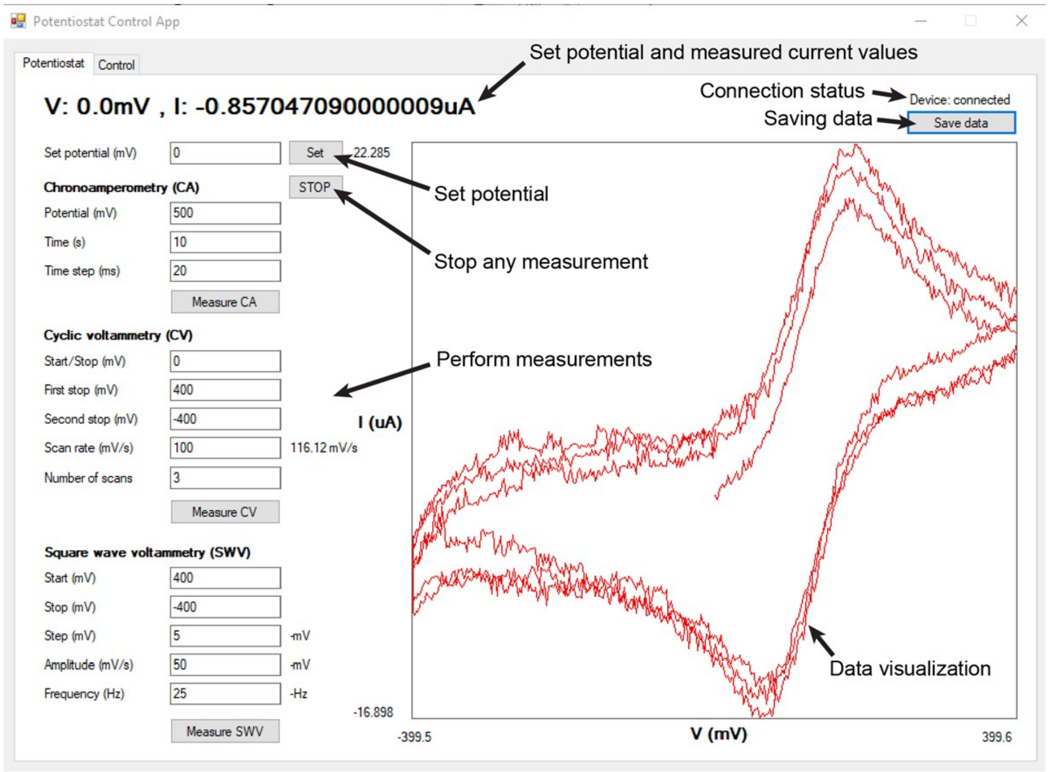

Figure S5. Main view of the Potentiostat Control Application.

2.5. Characterization of electrical performance

We characterized the electrical performance of the potentiostat in two steps, first potential control and thereafter the current measurement side.

2.5.1. Potential control

For calibration and characterization of the potential control we connected the counter and reference electrode together and to the one input terminal of the digital multimeter, while working electrode was connected to the other terminal of the multimeter. Fluke 8808A (5-1/2 digit) multimeter was used for the calibration. Multimeter has in the 2V range resolution 10µV, input resistance >10GΩ and maximum measurement uncertainty 0.46mV, while in the 20V range resolution is 100µV, input resistance 10MΩs and maximum uncertainty 2.1mV. Based on the measurements we determined a linear potential calibration (**Figure S6**) Electrode_Potential[V] =DAC_word*a+b with parameters described in **Table S4**.

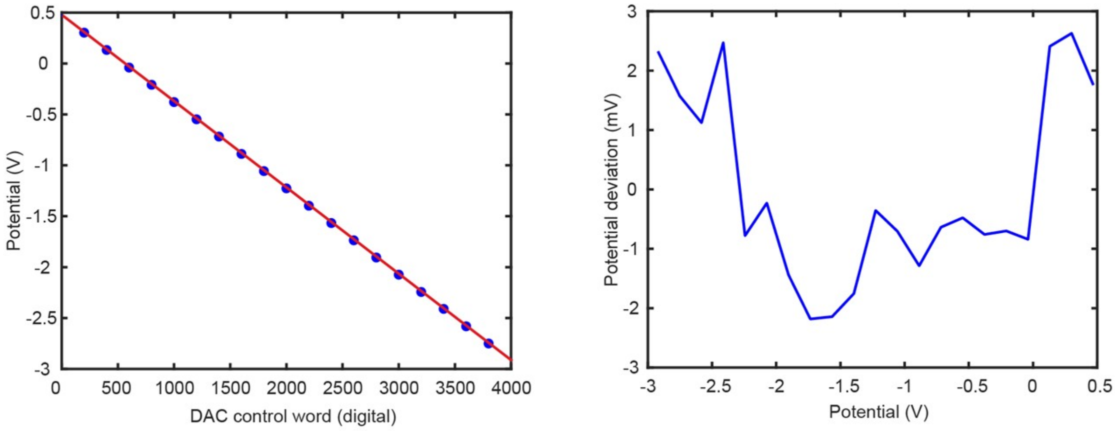

Figure S6. Potential calibration. Potential vs digital control word applied by potentiostat (left) and deviations from the linearity (right).

Table S4. Specification of the potential control. Uncertainties are given at confidence level approximately 68% (1 standard deviation).

**Parameter**	**Value**	**Unit**
Calibration slope (a) and resolution of the potential control	-0.84775±0.00029	mV/dig
Calibration offset (b)	469.69±0.69	mV
Minimum potential	-2.9	V
Maximum potential	0.47	V
Non-linearities (standard deviation)	1.6	mV
Maximum deviations in the full range	2.6	mV

2.5.2. Current measurement

Current measurements were performed by connecting 10kΩ resistor in between working electrode terminal and counter and reference electrode terminals connected together. Digital signal was recorded using the CV sweep, from which linear calibration constants were determined for the function Current[μA]=ADC_word*a+b, where calibration parameters can be found in **Table S5**. Open circuit noise was measured, when resistor was removed and working electrode terminal was disconnected (zero current). Calibration and test results are visualized on **Figure S7**.

Table S5. Specification of the current measurement. Uncertainties are given at confidence level approximately 68% (1 standard deviation).

Parameter	Value	Unit
Calibration slope (a) and resolution	2.8538±0.0019	nA/dig
Calibration offset (b)	-22.898±0.014	µA
Maximum negative current	-22	µA
Maximum positive current	12	µA
Non-linearities (standard deviation)	100	nA
Non-linearities (% of full scale)	0.29	%
Open-circuit noise	6.9	nA
Open-circuit noise (% of full scale)	0.02	%

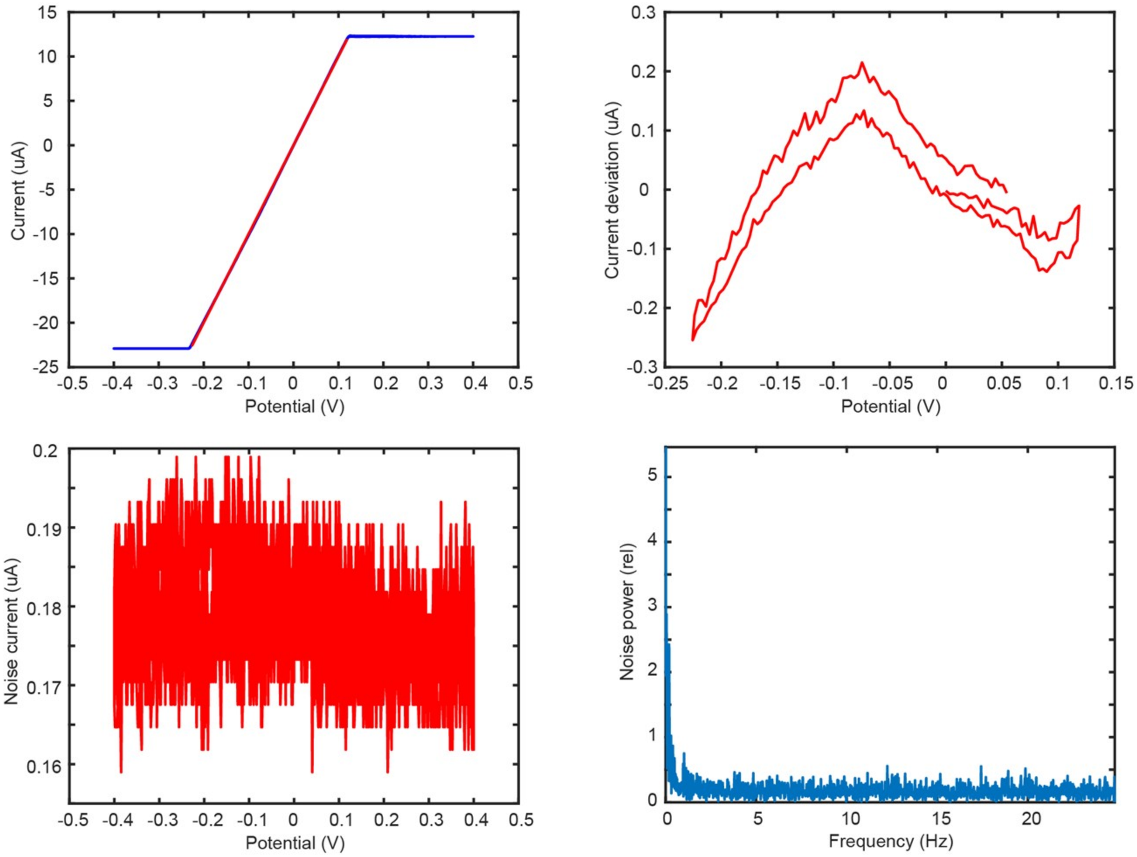

Figure S7. Current calibration (upper left), deviation from the linear calibration (upper right), open-circuit noise (lower left) and spectrum of the open-circuit noise (lower right). Noise was mostly white and no specific higher frequency component was observed in frequency rang <25Hz.

2.6. Additional Resources

A separate .zip file contains the following folders and files (source):

- Electronics – folder is containing the electronic design (Eagle)
    - Potentiostat.sch – Eagle circuit diagram
    - Potentiostat.brd – Eagle PCB board design (for manufacturing)
    - GerberFiles/ – Folder containing board design in Gerber format
- Firmware – folder is containing the Arduino firmware
    - Firmware.ino - Firmware for ATMEGA168
- App – folder is containing source code and compiled executable of Windows user interface application software (Visual Studio Project) to control the potentiostat.

2.7. References

(1) Alar Ainla, Maral P. S. Mousavi, Maria-Nefeli Tsaloglou, Julia Redston, Jeffrey G. Bell, M. Teresa Fernández-Abedul, George M. Whitesides, "Open-Source Potentiostat for Wireless Electrochemical Detection with Smartphones", *Anal. Chem*. 2018, 90, 10, 6240–6246, https://pubs.acs.org/doi/abs/10.1021/acs.analchem.8b00850

(2) https://github.com/alarainla/MiniPotentiostat

3. Figure S8

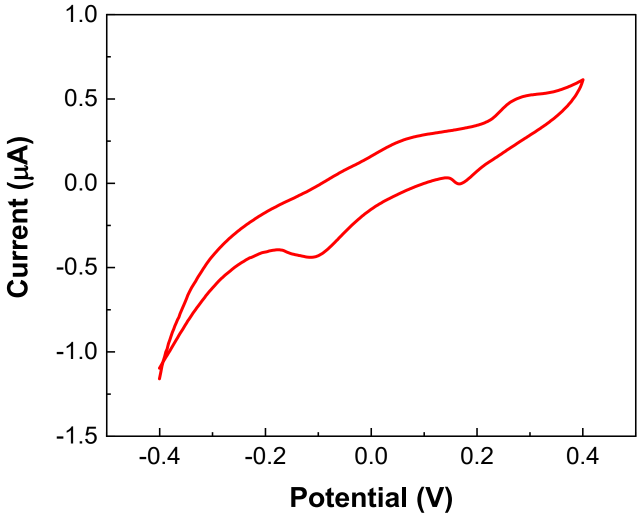

Figure S8. Representative CVs in TMB/H_2O_2 enzymatic substrate (scan rate of 30 mV/s) for gold electroplated electrodes after physisorption of TMB/H_2O_2.

4. Figure S9

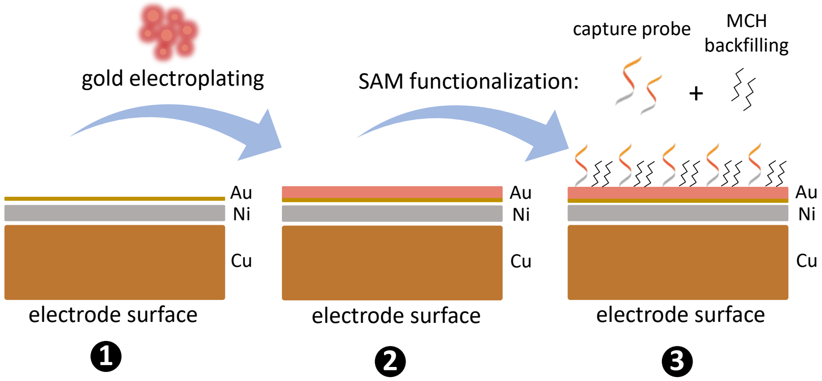

Figure S9. Cross section of the electrodes after: (i) standard manufacturing following PClass6 process for 2-layer PCB with Che Ni/Au surface finish; (ii) in-house gold electroplating; (iii) SAM functionalization with thiolated DNA capture probes and MCH backfilling.

5. Figure S10

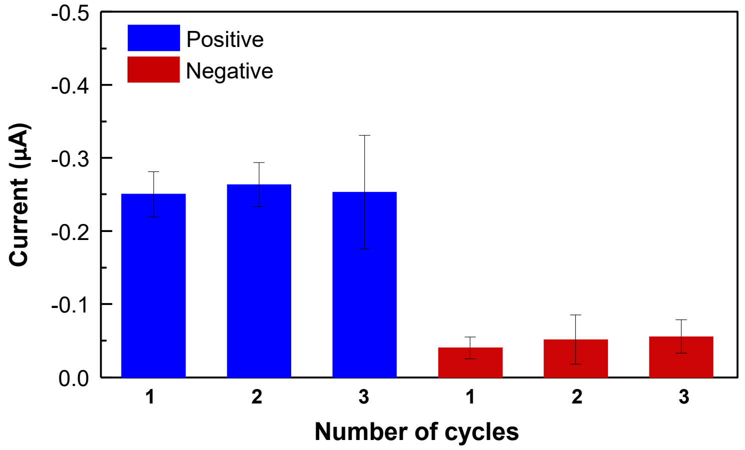

Figure S10. Electrochemical responses of the PCB-based electrochemical biosensor after 3 cycles of hybridization assay/electrochemical measurement/cleaning for gold working electrode 1 (WE1) using Autolab potentiostat. Error bars are the standard deviation of the mean (n = 3).
